# Privacy-preserving continual learning methods for medical image classification: a comparative analysis

**DOI:** 10.3389/fmed.2023.1227515

**Published:** 2023-08-14

**Authors:** Tanvi Verma, Liyuan Jin, Jun Zhou, Jia Huang, Mingrui Tan, Benjamin Chen Ming Choong, Ting Fang Tan, Fei Gao, Xinxing Xu, Daniel S. Ting, Yong Liu

**Affiliations:** ^1^Institute of High Performance Computing, Agency for Science, Technology and Research (A^*^STAR), Singapore, Singapore; ^2^Artificial Intelligence and Digital Innovation Research Group, Singapore Eye Research Institute, Singapore, Singapore; ^3^Duke-NUS Medical School, Singapore, Singapore; ^4^Singapore National Eye Centre, Singapore, Singapore

**Keywords:** continual learning, medical image classification, model deployment, optical coherence tomography, comparative analysis

## Abstract

**Background:**

The implementation of deep learning models for medical image classification poses significant challenges, including gradual performance degradation and limited adaptability to new diseases. However, frequent retraining of models is unfeasible and raises concerns about healthcare privacy due to the retention of prior patient data. To address these issues, this study investigated privacy-preserving continual learning methods as an alternative solution.

**Methods:**

We evaluated twelve privacy-preserving non-storage continual learning algorithms based deep learning models for classifying retinal diseases from public optical coherence tomography (OCT) images, in a class-incremental learning scenario. The OCT dataset comprises 108,309 OCT images. Its classes include normal (47.21%), drusen (7.96%), choroidal neovascularization (CNV) (34.35%), and diabetic macular edema (DME) (10.48%). Each class consisted of 250 testing images. For continuous training, the first task involved CNV and normal classes, the second task focused on DME class, and the third task included drusen class. All selected algorithms were further experimented with different training sequence combinations. The final model's average class accuracy was measured. The performance of the joint model obtained through retraining and the original finetune model without continual learning algorithms were compared. Additionally, a publicly available medical dataset for colon cancer detection based on histology slides was selected as a proof of concept, while the CIFAR10 dataset was included as the continual learning benchmark.

**Results:**

Among the continual learning algorithms, Brain-inspired-replay (BIR) outperformed the others in the continual learning-based classification of retinal diseases from OCT images, achieving an accuracy of 62.00% (95% confidence interval: 59.36-64.64%), with consistent top performance observed in different training sequences. For colon cancer histology classification, Efficient Feature Transformations (EFT) attained the highest accuracy of 66.82% (95% confidence interval: 64.23-69.42%). In comparison, the joint model achieved accuracies of 90.76% and 89.28%, respectively. The finetune model demonstrated catastrophic forgetting in both datasets.

**Conclusion:**

Although the joint retraining model exhibited superior performance, continual learning holds promise in mitigating catastrophic forgetting and facilitating continual model updates while preserving privacy in healthcare deep learning models. Thus, it presents a highly promising solution for the long-term clinical deployment of such models.

## 1. Introduction

Continual learning refers to the process of continually training and updating a deep learning model over time, as new data becomes available. This approach is particularly pertinent for medical image classification model deployment because any potentially deployed deep learning model could suffer from a gradual decline in performance from underlying distribution shifts over time. By continuously retraining the model with new data, the model could maintain high classification performance and adapt to changes in the data distribution. However, in continual learning scenarios, the conventional deep learning approach often leads to catastrophic forgetting, where the model experiences memory loss or a significant decline in performance on previous classes after being trained on new tasks or datasets (1). This is a commonly reported phenomenon in deep learning because the model prioritizes its weights and biases optimization for the new task, leading it to forget or overwrite previously learned information. Alternatively, to mitigate catastrophic forgetting in medical image classification, one potential solution is to retrain the model with cumulative data whenever a new dataset becomes available. However, retraining from scratch frequently in the model's deployment phase is not practical. Furthermore, data privacy is of utmost importance in the medical domain, and due to strict regulatory requirements, it may not always be possible to access old data (2). Additionally, medical data is often stored in dedicated servers, making it difficult to shuffle multiple datasets. Researchers have proposed a number of continual learning approaches to overcome catastrophic forgetting. However, there have been few studies on medical imaging using continual learning. Furthermore, achieving a trade-off between stability and plasticity remains another challenge in the continual learning scenario. Stability refers to retaining previously acquired knowledge, while plasticity pertains to the model's ability to learn new knowledge from the new data.

In the context of continual learning, “non-storage” refers to the absence of storing or retaining old data from previous tasks or classes. Continual learning approaches can be categorized into two broad groups: exemplar-based and exemplar-free approaches. Exemplar-based approaches store a small number of data or exemplars and reintroduce them with the new data during training to prevent the model from forgetting the old knowledge. Conversely, exemplar-free approaches (non-storage) rely on regularization, expansion, and generative replay to achieve similar goals without storing exemplar data. Given concerns about privacy and accessibility with medical data, the storage of previous exemplar data from old studies is not feasible. Therefore, exemplar-free continual learning approaches are preferable for medical image classification models and preserve healthcare privacy.

The training process for continual learning happens sequentially in a series of tasks, and during each training session, the model only has access to the data for the current task. *Task incremental, domain incremental* and *class incremental* are three common scenarios of continual learning. In task incremental learning, the model has access to the task identifier during inference, which obviates the need for the model to differentiate between images from different tasks. Domain incremental learning, on the other hand, does not require task identification at inference time, since the output space of each task is the same. Class incremental learning, the most clinically relevant yet challenging scenario, involves the model's inability to access the task identifier at inference time, and the model must therefore be capable of distinguishing between images from all tasks. The class incremental learning is more closely aligned with real-life scenarios and more suitable for real-world medical image classification.

Hence, in this research, privacy-preserving exemplar-free continual learning approaches were explored in class learning scenarios for medical imaging classification targeting significant and prevalent diseases using publically available datasets, namely optical coherence tomography (OCT) (3) and PathMNIST (4). As a benchmark for continual learning performance, CIFAR10 dataset (5) was included.

## 2. Background

Continual learning involves training machine learning models on data from a series of tasks *D* = {*D*_1_, *D*_2_, ⋯ , *D*_*T*_}. Task $D_{t}={\left\{\left(x_{i}^{t},y_{i}^{t}\right)\right\}}_{i=1}^{n_{t}}$ is the *t*^*th*^ task where $x_{i}^{t}\in X_{t}$ is an input, $y_{i}^{t}\in Y_{t}$ is the corresponding label and *n*_*t*_ is number of classes in the task. During training the *t*^*th*^ task, only training data *D*_*t*_ is available while the training data of previous tasks are no longer accessible. The tasks are generally assumed to be distinct from one another. The goal of continual learning is to train a parameterized model *f*_θ_:*X*→*Y* that can predict the correct label for an unseen test sample from any task. Here $X=\cup_{t=1}^{T}X_{t}$ is the input space and $Y=\cup_{t=1}^{T}Y_{t}$ is the output space. In continual learning, the marginal probability distribution of inputs varies across tasks, i.e., *P*(*X*_1_)≠*P*(*X*_2_). Based on probability distribution of output space and whether the task identity is provided at the inference time, there are three different continual learning scenarios (6).

### 2.1. Task incremental scenario

The probability distribution of output space varies between tasks (*P*(*Y*_1_)≠*P*(*Y*_2_)) and task identifier is provided at the time of inference for task incremental scenario. Hence, it is possible to train models with task-specific components in this scenario. “Multi-headed” network architecture is commonly used for this scenario where each task has its own output units, but the rest of the network is shared among tasks.

### 2.2. Domain incremental scenario

The output space (and hence the corresponding probability distribution) remains same across the tasks for domain incremental scenario, i.e., {*Y*_1_} = {*Y*_2_} and *P*(*Y*_1_) = *P*(*Y*_2_). The problem setting looks similar to domain adaptation (7). However unlike domain adaptation, which focuses on achieving good performance on new task, the goal of continual learning is to maintain good performance on previously learned tasks while also achieving reasonable performance on new tasks.

### 2.3. Class incremental scenario

**Figure 1:**
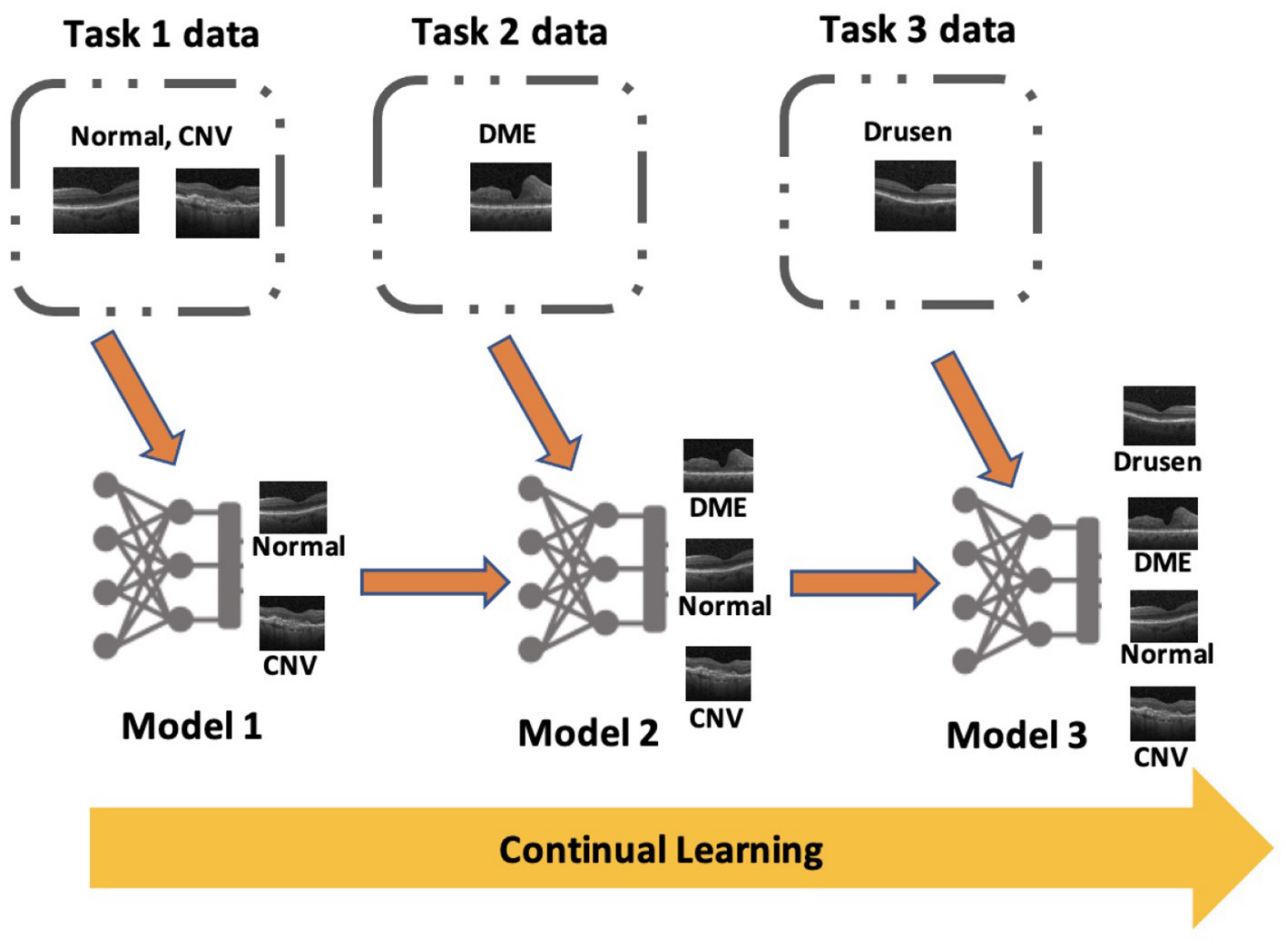
An example for class incremental scenario in medical imaging classification: Continual learning based deep learning algorithm sequentially learns a series of retinal pathologies in OCT such as CNV, DME, Drusen, and normal finding.

Similar to task incremental scenario, the probability distribution of output space varies between tasks in class incremental scenario. However, the model does not have access to task identifier at the time of inference which make it the most complex scenario of continual learning. The network architecture for class incremental scenario is generally “single-headed” where a single output layer is used to make predictions for all tasks. Sometimes “multi-headed” architectures are used for this scenario, but it needs prediction of task identifier before predicting the class label of the image at the time of inference. Figure 1 shows an example of a “single-headed” model which is being trained in class incremental scenario to classify OCT images into different retinal pathologies. In many real-world applications, it is not practical to assume that the task identifier will be available at the time of inference, especially in the medical domain where the model is often used to classify diseases. Therefore, an exclusive focus was placed on this scenario in this research.

## 3. Exemplar-free continual learning approaches

In this section, three main categories of exemplar-free continual learning approaches and associated algorithms were summarized.

### 3.1. Regularization-based methods

Regularization-based continual learning methods add a regularization term to the training loss function that encourages it to retain the knowledge it has learned from previous tasks while also allowing it to adapt to new tasks. Regularization-based methods can be further divided into weight-based and data-based regularization methods.

The first group of regularization-based approaches aims to prevent weight drift, which is considered to be crucial for previous tasks. This is achieved by estimating the importance of each parameter in the network after learning each task. When training new tasks, the importance of each parameter is taken into account and used to discourage its changes. For example, Elastic Weight Consolidation (EWC) (8) uses a quadratic penalty term to restrict modification of important weights. (9) proposed a quadratic penalty method for continual learning of neural networks that contain batch normalization layers. Synaptic Intelligence (SI) method (10) uses synapse to measure the weights' importance. Memory Aware Synapses (MAS) (11) determines the importance of weights using a Hebbian learning model, which is based on the sensitivity of the output function. Riemanian Walk (RWalk) (12) method uses Fisher Information Matrix approximation and online path integral to calculate the importance for each parameter. (13) defined a notion of uncertainty and made the variance of the incoming weights of each node trainable. To maintain stability-plasticity trade-off, they also included two regularization terms for stability and plasticity respectively. Similarly, (14) added a drifting regularization for stability and a Lasso regularization for plasticity. There are a few methods which regularize gradients of weights. For instance, Orthogonal Weights Modification (OWM) (15) maps the weights modification (gradients) onto a subspace generated by all the previous tasks in order to maintain the performance of previous tasks. Gradient Projection Memory (GPM) (16) regularizes the gradients by restricting the direction of gradient descent steps. Likewise, (17) proposed conceptor-aided backpropagation (CAB), in which gradients are shielded by conceptors (characterizes the linear subspace formed by activation in a layer) against degradation of previously learned tasks.

On the other hand, data-based regularization methods use knowledge distillation (18) to prevent the model from forgetting. Knowledge distillation refers to the technique of transferring the knowledge of a model trained on previous tasks to a new model that will learn new tasks. The basic idea is to use the output of the model trained on previous tasks as a “soft target” for the new model. The new model is trained to mimic the output of the old model on the previous tasks, as well as learn new tasks. Learning without Forgetting (LwF) (19) is one of the earliest methods to use knowledge distillation for continual learning. Extending on LwF, (20) proposed Learning without Memorizing (LwM), which adds attention distillation loss by Gradient-weighted Class Activation Mapping (Grad-CAM) along with knowledge distillation loss to mitigate catastrophic forgetting. There are a few methods such as PODNet (21), Attention Uncertainty (AU) (22) and GeoDL (23) which incorporate exemplar memory in addition to knowledge distillation techniques to address the challenge of continual learning.

A major drawback of regularization-based approaches is that they are highly sensitive to the selection of hyperparameters, making it challenging to achieve a suitable balance between stability and plasticity.

### 3.2. Expansion-based methods

In contrast to regularization-based methods, expansion-based continual learning approaches focus on expanding the capacity of the model to handle new tasks. This can be achieved by adding new parameters or neurons to the model, or by creating multiple versions of the model, each specialized for a specific task.

One popular approach of expansion-based continual learning is called dynamic expansion, where the model's capacity is expanded by adding new neurons or parameters to the model as new tasks are encountered. This approach allows the model to adapt to new tasks by creating new representations or features that are specific to the new task. Dynamically Expandable Network (DEN) (24), Reinforced Continual Learning (RCL) (25) and Compacting Picking Growing (CPG) (26) are some methods which fall under this category.

Another approach is called task-specific expansion, where either multiple versions of the model (each specialized for a specific task) are created, or task specific classifier is added to the model. This allows the overall system to handle multiple tasks simultaneously. Progressive Network (27), Additive Parameter Decomposition (APD) (28), Efficient Feature Transformation (EFT) (29) and Expert Gate (30) are some of the methods that utilize task-specific expansion to continuously learn new tasks without forgetting previous tasks.

Most of the existing expansion-based methods are suitable for the task-incremental scenario, i.e., they assume availability of task identity at the time of inference. However, certain methods such as Expert Gate, iTAML and EFT predict the task identifier prior to predicting the correct class. Expansion-based methods typically exhibit superior performance compared to regularization-based methods. However, the requirement for two levels of inferences, first determining the task identity and then the actual class label, may decrease the overall performance of the model.

### 3.3. Generative replay-based methods

The idea of generative replay-based methods in continual learning is to generate synthetic data that resembles the distribution of old tasks, and use them to train the model along with the data in new tasks. This allows the model to learn new tasks while maintaining knowledge of previously learned tasks, without the need to keep any exemplars from those tasks. Some popular generative methods used in continual learning include generative replay (GR) (31), Replay-through-Feedback (RtF) (32) and Brain Inspired Replay (BIR) (33). GR utilizes a generative adversarial network to generate previous data whereas RtF and BIR use a variational autoencoder as generator.

The main disadvantage of generative method is that it takes a long time to train the generative model. Furthermore, it has been shown that generative models may struggle when dealing with complex datasets (34).

## 4. Compared methods

In this study, pertinent algorithms from each category of exemplar-free continual learning methods were selected. Within the weight-based regularization methods category, EWC (8), SI (10), MAS (11), MUC-MAS (35), RWalk (12), OWM (15), and GPM (16) were included. For data-based regularization, LwF (19) and LwM (20) were selected. EFT (29) was chosen as the expansion-based method, while GR (31) and BIR (33) were selected from the generative-replay methods. The lower baseline (finetune) and the upper baseline (joint) approaches were included for comparison. Finetuning involves adapting a pre-trained model to new data or tasks without starting from scratch, while joint training relies on retraining the model on cumulative data from all tasks. A comprehensive description of these selected algorithms is provided in the Supplementary material.

## 5. Methodology

The model underwent incremental training on three tasks. By definition, Model 1 was initially trained on the first task, followed by expanding Model 1 into Model 2 through sequential training on task 2 data. Similarly, Model 2 was further expanded into Model 3 after being trained on task 3 data. The detailed network architecture and hyperparameter values are provided in the Supplementary material.

### 5.1. Datasets

Two medical datasets: optical coherence tomography (OCT) (3) and PathMNIST (36) and one non-medical dataset: CIFAR10 (5) were selected. All images are in RGB color, normalized using the mean and standard deviation of ImageNet. All three datasets were split into three tasks and the set of classes in each task was fixed across all experiments. The specific class description for each dataset is specified below.

- **OCT:** This dataset contains over 108,309 publicly available OCT training images, including four classes regarding the condition of the retina: Normal (47.21%), Drusen (7.96%), Choroidal Neovascularization (CNV) (34.35%), Diabetic Macular Edema (DME) (10.48%). There are 250 testing images for each class. The first task contains two classes: Normal and CNV, the second task contains only DME and the last task contains only Drusen. It was selected for its highly imbalanced classes simulating a more realistic continual learning scenario, particularly when the model has already been trained on a large number of data in earlier tasks and the new task only contains a small number of training images. Furthermore, additional investigations on continuous learning sequences were explored for the significance of unbalanced data distribution, as reported in the Supplementary material. Those scenarios include task 1 containing a large amount of data while task 3 containing the least amount of data, and vice versa.
- **PathMNIST:** As part of MedMNIST dataset, PathMNIST dataset is selected due to its distinguishing feature of encompassing a greater number of disease classes. It consists of histology slides with 9 different colon pathology classes. It contains 89,996 training and 7,180 testing images. The number of training images in a class varies from 7,886 to 12,885, with an average of 10,000. The 9 classes are divided into three tasks containing three classes each.
- **CIFAR10:** As a traditional continual learning benchmark, CIFAR10 dataset was selected to compare the adaptability and robustness of different algorithms. It consists of 60,000 natural images in 10 classes, such as cat and truck. There are 5,000 training images and 1,000 testing images per class. The first task contains four classes while the two subsequent tasks contain three classes each.

### 5.2. Statistical analysis

Experiments were conducted using three different seed values. Following common practices in the field of continual learning (12, 37), average accuracy and average forgetting were selected as the evaluation metrics. Average accuracy measures the overall performance of the model after training on task *t* is complete. It is computed as $A_{t}=\frac{1}{t}\sum_{i=1}^{t}a_{t}^{i}$, where $a_{t}^{i}$ is the accuracy of the model on task *i* after training on task *t*. On the other hand, task-wise accuracy was also introduced to highlight model adaptation and the balance between stability and plasticity during sequential training, measured by the intermediate and final accuracies of each task on the intermediate (Model 1 and Model 2) and final model (Model 3). The forgetting metric was included by measuring the decline in accuracy for each task by comparing the highest accuracy achieved during training with the final accuracy after training is completed. This provides an estimate of the extent model has been forgotten based on its current state. The forgetting on task *i* after the model has been trained on task *t* is given by $f_{t}^{i}=\underset{j\in \left\{1,\cdots \;,t-1\right\}}{\max}\left(a_{j}^{i}-a_{t}^{i}\right)$. The average forgetting of the final model is computed as $F=\frac{1}{T-1}\sum_{i=1}^{T-1}f_{T}^{i}$.

## 6. Results

The average accuracy and forgetting of the final model (Model 3) after it has been trained on the three tasks sequentially are calculated. The task-wise accuracy on each model is also measured for a better insight into the balance between stability and plasticity.

### 6.1. Average accuracy and forgetting

**Figure 2:**
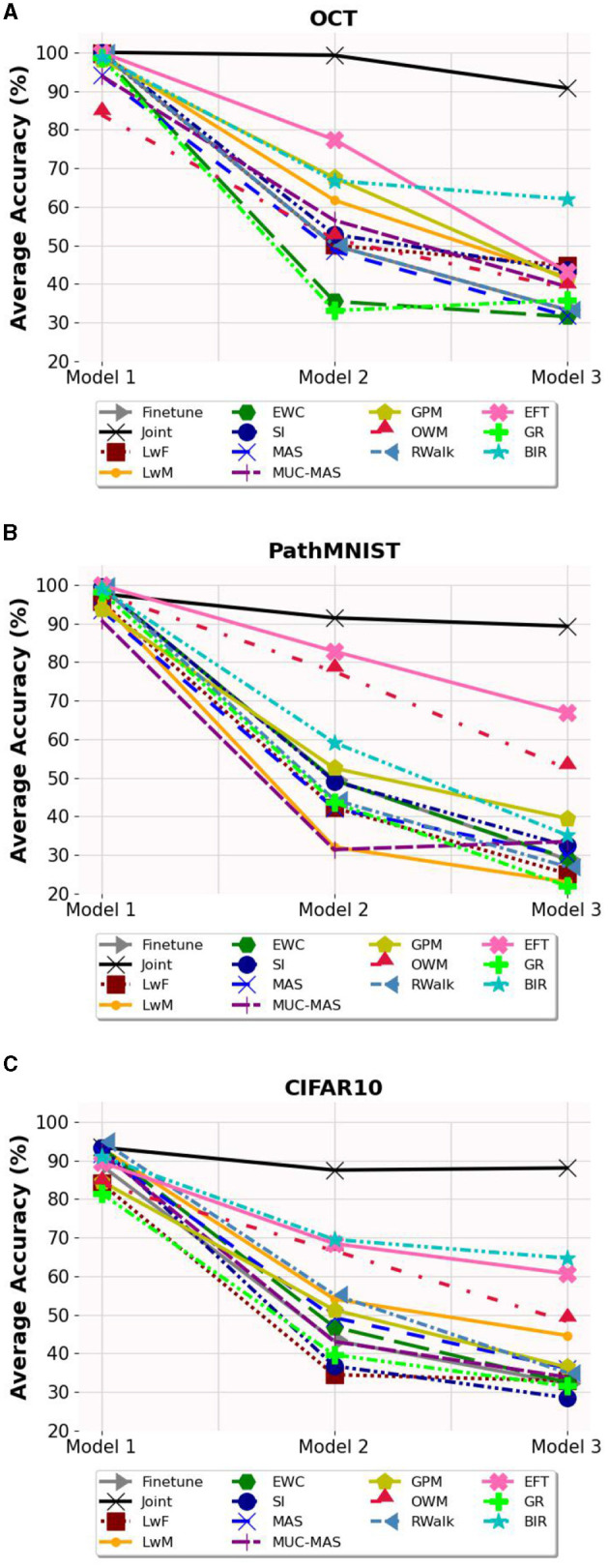
The average accuracy on the three models. **(A)** Average accuracy on OCT dataset. **(B)** Average accuracy on PathMNIST dataset. **(C)** Average accuracy on CIFAR10 dataset.

The average accuracy and forgetting of Model 3 are presented in Table 1, and the effect of sequential training on the average accuracy of the model is shown in Figure 2. For average accuracy, joint training obtains best performance of 90.76%, 89.28% and 88.01% respectively and it serves as the upper bound. Among all validated algorithms, BIR shows the best retinal disease classification on OCT and CIFAR10 performance with average accuracy of 62.00% (95% CI 59.36-64.64%) and 64.68% (95% confidence interval (CI) 63.76-65.59%) respectively. EFT obtains the best colorectal cancer histology classification on PathMNIST with average accuracy of 66.82% (95% CI 64.23-69.42%). Additionally, EFT demonstrates consistent better accuracy and lower forgetting across different datasets with average accuracy of 43.20% (95% CI 41.24-45.16%) on OCT and 60.65% (95% CI 58.57-62.73%) on CIFAR10. RWalk's performance is poor and is comparable to the performance of Finetune with catastrophic forgetting. Other algorithms such as LwF, EWC, MAS, MUC-MAS and GR appear to have comparable performance to Finetune in terms of average accuracy. However, they are still able to retain some knowledge of previous tasks (evident from their lower forgetting), in contrast to Finetune, which almost completely forgets previous tasks, as can be observed in Table 2. GR performs poorly on all datasets in contrast to similar generative replay method BIR.

**Table 1:** Average accuracy and forgetting of Model 3 for the three datasets.

**Category**	**Method**	**OCT**		**PathMNIST**		**CIFAR10**	
		**Accuracy**	**Forgetting**	**Accuracy**	**Forgetting**	**Accuracy**	**Forgetting**
Baseline	Finetune	33.33	100	28.89	99.18	32.20	86.47
	Joint	90.76	-	89.28	-	88.01	-
Regularization	LwF	44.8	80.23	25.20	81.33	32.90	65.62
	LwM	41.75	41.58	23.02	35.43	44.66	41.48
	EWC	31.54	76.83	29.16	95.82	32.53	93.32
	SI	43.60	**21.27**	32.40	28.94	28.51	40.62
	MAS	31.73	83.69	29.97	74.54	36.18	80.07
	MUC-MAS	39.32	76.87	33.49	52.19	33.84	65.10
	GPM	41.16	26.41	39.51	24.96	36.47	36.18
	OWM	38.93	83.10	52.42	**16.34**	48.30	42.18
	RWalk	33.33	100	27.05	85.13	35.00	90.99
Expansion	EFT	43.20	38.13	**66.82**	29.39	60.65	**31.31**
Generative	GR	35.83	66.05	21.95	90.88	31.50	74.28
	BIR	**62.00**	51.31	35.17	88.17	**64.68**	42.30

The bold values indicate best results for each column.

**Table 2:** Task wise accuracy of Model 3 for the three datasets.

**Category**	**Method**	**OCT**			**PathMNIST**			**CIFAR10**		
		**Task 1**	**Task 2**	**Task 3**	**Task 1**	**Task 2**	**Task 3**	**Task 1**	**Task 2**	**Task 3**
Baseline	Finetune	0.0	0.0	100	0.0	0.0	86.67	0.0	2.53	94.09
	Joint	99.47	97.47	75.33	95.99	85.93	85.92	86.03	86.47	91.55
Regularization	LwF	0.53	39.33	94.53	0.63	15.09	59.83	0.14	21.76	76.79
	LwM	63.13	0.93	61.20	28.97	26.69	13.38	36.65	26.81	73.51
	EWC	0.01	0.13	94.47	1.01	5.20	81.27	0.01	0.01	97.57
	SI	63.33	14.53	52.93	49.36	24.84	22.99	29.85	0.42	55.26
	MAS	0.13	10.37	84.68	2.04	6.07	81.78	3.35	18.05	87.16
	MUC-MAS	9.28	20.99	87.69	18.81	4.14	77.51	5.58	18.69	77.26
	GPM	90.66	1.73	31.07	79.77	2.23	36.53	58.1	4.0	47.30
	OWM	14.40	13.60	88.80	77.72	73.18	6.35	32.35	49.61	62.94
	RWalk	0.0	0.0	100	0.22	0.06	80.88	0.81	6.33	97.86
Expansion	EFT	61.47	38.40	29.73	59.60	73.63	67.24	41.10	60.74	80.10
Generative	GR	2.57	16.81	88.11	0.26	1.82	63.77	2.74	7.53	84.22
	BIR	10.53	78.40	97.07	17.33	3.38	84.82	30.18	68.13	95.71

In terms of forgetting, although the accuracy of SI is not high, it demonstrates the least forgetting (21.27% on OCT). For PathMNIST and CIFAR10 datasets, OWM and EFT show the least forgetting, measured by 16.34% and 31.31% respectively. We also note that the performance of exemplar-free continual learning methods is greatly influenced by the structure of the dataset. For instance, OWM, which performs relatively well on PathMNIST (52.42% accuracy, 16.34% forgetting) and CIFAR10 (48.30% accuracy, 42.18% forgetting), does poorly on the unbalanced data of OCT (38.93% accuracy, 83.10% forgetting). In contrast, SI performs better on OCT (43.6% accuracy, 21.27% forgetting) and performs relatively worse on PathMNIST (32.40% accuracy, 28.94% forgetting) and CIFAR10 (28.51% accuracy, 40.62% forgetting).

In summary, EFT and BIR exhibit better overall accuracy compared to the other selected exemplar-free continual learning methods.

### 6.2. Task-wise accuracy

**Figure 3:**
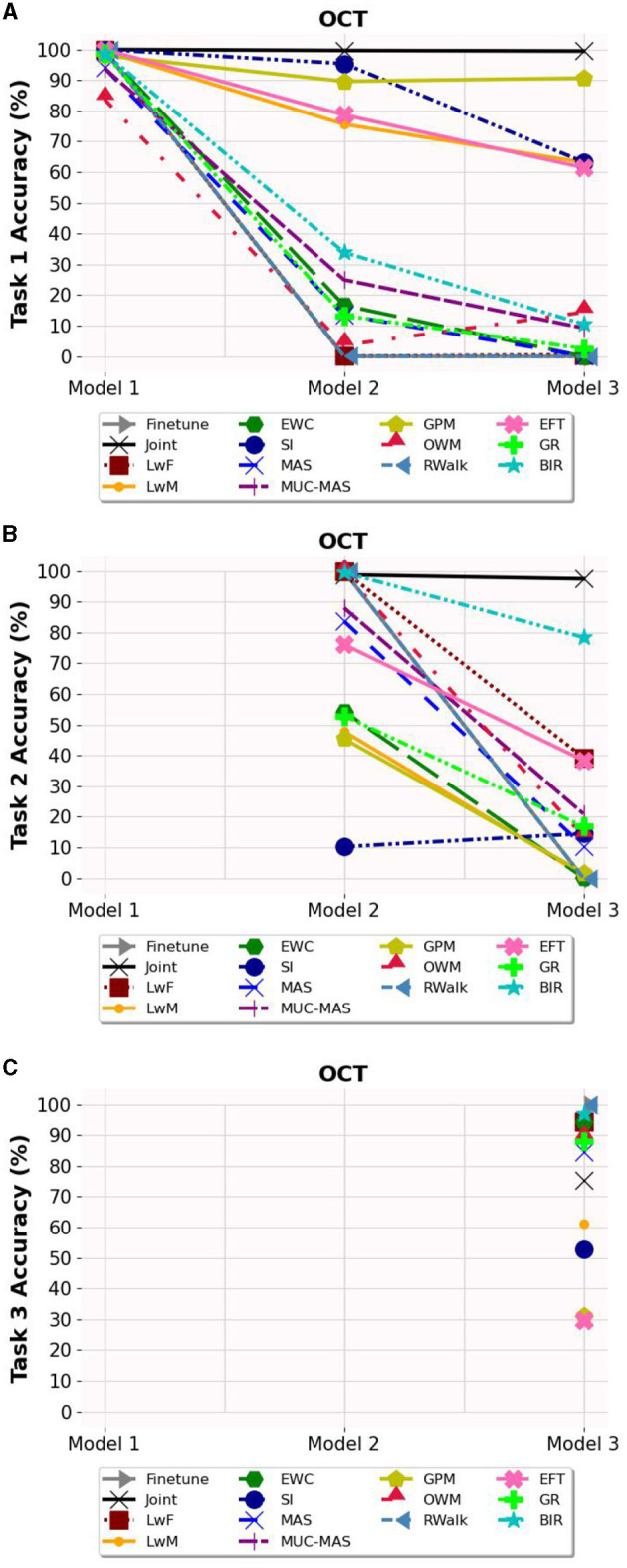
Task wise accuracy of OCT dataset on the three models. **(A)** Task 1 accuracy. **(B)** Task 2 accuracy. **(C)** Task 3 accuracy.

**Figure 4:**
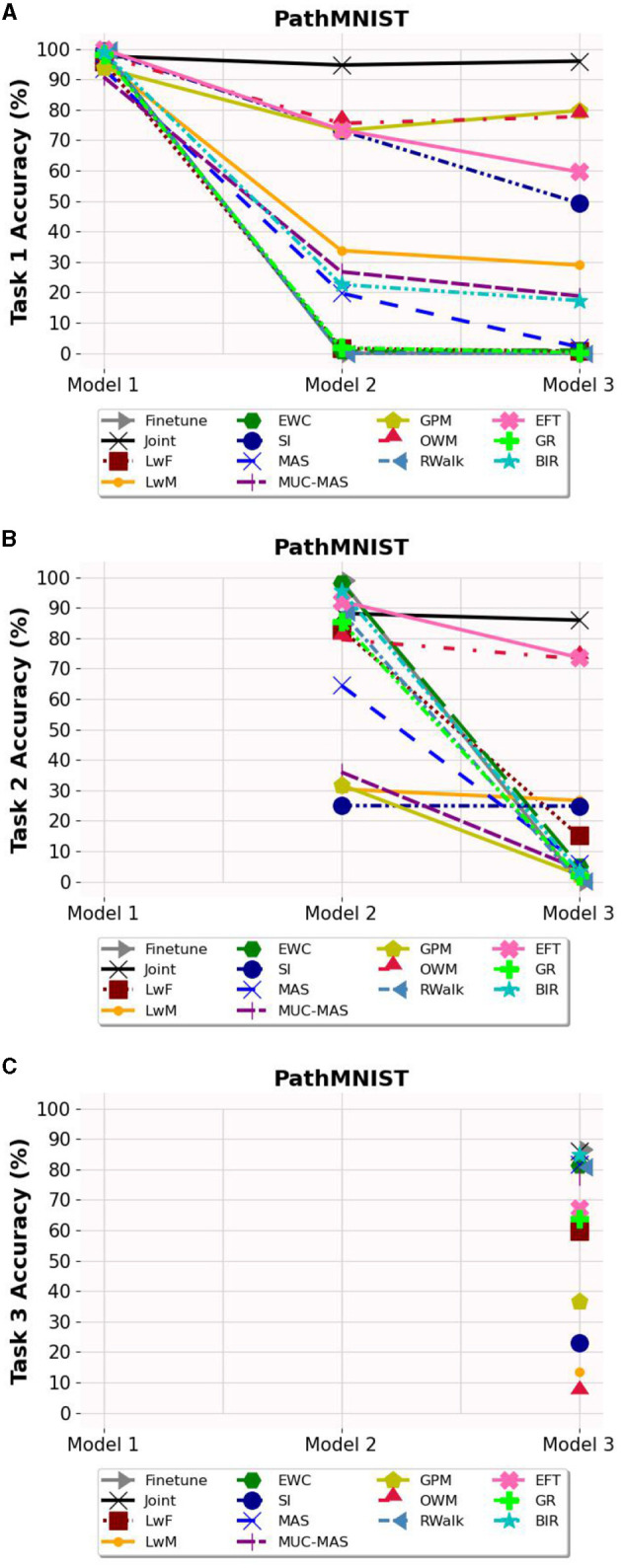
Task wise accuracy of PathMNIST dataset on the three models. **(A)** Task 1 accuracy. **(B)** Task 2 accuracy. **(C)** Task 3 accuracy.

**Figure 5:**
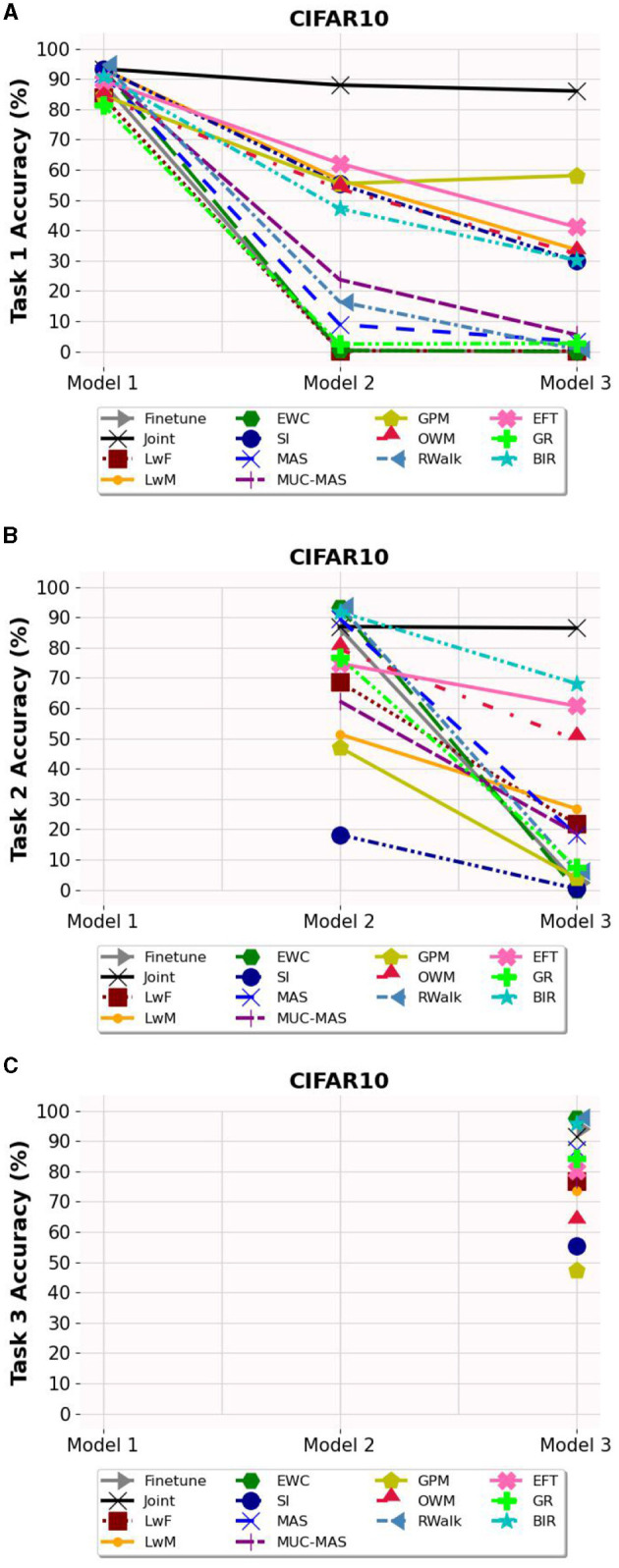
Task wise accuracy of CIFAR10 dataset on the three models. **(A)** Task 1 accuracy. **(B)** Task 2 accuracy. **(C)** Task 3 accuracy.

Based on Figures 3–**5**, the accuracy of each task is affected after the model is trained on each task for OCT, PathMNIST and CIFAR10 datasets respectively. Plots in Figures 3A, 4A, 5A show the decline in accuracy of task 1 data after sequential training on the three tasks. The X-axis represents the three models. It is observed that although the initial accuracy on task 1 is almost similar for all methods, it drastically declines (except for a few well-performing methods such as EFT, BIR, etc.) after training on task 2. However, the accuracies of task 2 data vary widely (Figures 3B, 4B, 5B) depending on the stability-plasticity trade-off used by each method. The initial accuracy of task 2 for methods such as SI, LwM, and GPM is relatively low as, to maintain stability, the model is not flexible enough to incorporate new knowledge. The same behavior is observed for the task 3 data (Figures 3C, 4C, 5C), and the initial accuracy of task 3 data varies with the algorithm.

Results reported in Table 2 provides a better picture of the stability-plasticity trade-off employed by each algorithm where the accuracy of each task on Model 3 was reported. Algorithms such as LwF, EWC, MAS, RWalk and GR almost completely forget previous tasks and their average accuracy reported in Table 1 is mainly due to the high accuracy of task 3. Based on the relatively better performance of EFT across three different datasets, it supports the assertion that expansion-based methods, which add task-specific nodes to the model, are better able to retain the knowledge of previous tasks compared to regularization-based methods. As EFT maintains a task-specific classifier, it is able to maintain relatively better accuracies on each task.

It is noteworthy that for some algorithms such as LwM, SI, and GPM, the final accuracy of task 1 is higher than the final accuracy of task 2, which appears counterintuitive. However, as seen in Figures 3B, 4B, 5B, to maintain stability, the initial accuracies of task 2 are low to begin with. Therefore, when the model is subsequently trained on task 3, the accuracy of task 2 further declines and is lower than the final accuracy of task 1 (which was high to begin with). This explains the behavior of having task 2's final accuracy lower than task 1's final accuracy.

The majority of methods, except for EFT and OWM to some extent, exhibit poor performance on the PathMNIST dataset. This could be attributed to the presence of overlapping features among classes across different tasks. The limited success of OWM on this dataset highlights the existence of feature overlap, as even with an extensive training regime of 500 epochs and a learning rate of 1e7, OWM struggled to identify a suitable subspace in the feature space for the third task, despite its attempt to project the gradients of the new task orthogonally to the subspace of previous tasks.

Another notable observation is that while methods like LwF and BIR excel at retaining knowledge of the most recent task, they tend to exhibit a higher degree of forgetting as the number of tasks increases. For instance, although BIR demonstrates the highest average accuracy in both OCT and CIFAR10, it still experiences significant forgetting (51.31% on OCT and 42.30% on CIFAR10). This indicates that although these models achieve high accuracy on current tasks, they struggle to retain information about earlier tasks. Additionally, regularization-based methods are highly sensitive to hyperparameters, and even small changes to the regularization loss coefficient can lead to vastly different results.

In the context where the later tasks have more training data compared to the earlier tasks, BIR consistently maintains its performance on the OCT dataset, even when the class sequence is altered. This is demonstrated in the Supplementary material. However, while EFT maintains its overall accuracy, its task-wise accuracy exhibits variations for different sequences. GR's performance remains unaffected by data imbalance; however, its overall accuracy remains low, and forgetting remains high. Other regularization-based methods, including LwF, EWC, MAS and MUC-MAS demonstrate similar patterns. For the remaining regularization-based methods, such as LWM, SI, GPM, OWM and RWalk changes in the task sequence impact their task-wise accuracy. Despite this, their overall accuracy remains low.

In conclusion, striking a balance between stability and plasticity poses a significant challenge, especially for regularization-based algorithms. However, generative-based and expansion-based methods show promise in enhancing classification accuracy for tasks in the context of continual learning. Furthermore, generative-based methods are well-suited for scenarios where data is imbalanced across tasks. These approaches hold the potential for addressing the complexities of retaining previously learned knowledge while accommodating new information.

## 7. Discussion

In the field of medical imaging, deep learning models have been widely used for classification tasks across various medical imaging modalities. These models have been incorporated into real-world practice for decision-making in diagnosis and treatment, as evidenced by recent examples such as referable diabetic retinopathy screening in ophthalmology using color fundus photography (38). While deep learning models have provided a balance between healthcare burden and disease management, emerging imaging devices and new disease pathologies require further improvement of existing models. Continual learning has the potential to mitigate catastrophic forgetting and enable continual model updates, making it a promising solution for medical image classification models. In our study, we focused on comparing exemplar-free continual learning methods for medical image classification. We observed that regularization-based methods generally struggled in addressing catastrophic forgetting, while expansion-based methods and generative replay-based methods showed potential in retaining knowledge of earlier tasks. Although the AI field frequently reports on objective metrics like forgetting, it lacks clinical reliability and applicability. This is primarily due to its limited inter-model variability and weak correlation with model performance as demonstrated. Additionally, as the concept of continual learning is still in its early stages within the medical field, determining the average accuracy threshold for clinical deployment remains an unexplored area that necessitates careful consideration of balancing healthcare privacy concerns.

Our results show a considerable gap between best-performing continual learning algorithms with traditional joint training model which involves storing previous data and retraining the model from scratch. However, this conventional joint training model would not exist in the real world due to its storage for retraining strongly violating healthcare patient privacy. Despite challenges for current continual learning-based methods to achieve optimal classification performance, continual learning based deep learning model would likely be the next paradigm for medical image classification models with the aforementioned advantages. Another interesting topic is the balance between loss in the deep learning model's performance and breakage in individual patient privacy. As the next paradigm for deep learning in medical imaging, continual learning could potentially offer cross-institutional training for expanding model generalizability, learning about new diseases, and even enhancing limited data on rare diseases. Federated Learning (FL) (39) offers an alternative to deep learning when dealing with imbalanced healthcare data and healthcare privacy issues, relying on distributed localized model training and subsequent updating centralized models without exchanging raw input data. This approach has shown practical applicability in real-world scenarios, particularly in multi-center collaborations focused on COVID-19 detection during the recent pandemic (40) during the last pandemic. While continual learning may appear similar to FL, its distinct strength lies in the fact that knowledge is “memorized” within the model parameters, eliminating the need for complex adjunct security measures like differential privacy or blockchain integration in FL. Moreover, in FL, all the training data is assumed to be available simultaneously, albeit on different local clients. On the other hand, continual learning deals with the temporal factor, where training data arrives with time, and older training data may become unavailable due to privacy concerns.

As for continual learning techniques, although many original continual learning approaches achieve their best performance in task incremental scenarios, class incremental scenarios are more realistic in healthcare settings and continual learning deployment in medical image classification due to intrinsic input data complexity and uncertainty to task information. However, not all existing continual learning algorithms perform well in the class incremental learning scenario, due to their unique design and inconsistent performance in different seeds. Additionally, certain algorithms demonstrated robustness in handling data imbalance across tasks. The strength of our research lies in being the first comparative study that extensively analyzed all existing privacy-preserving continual learning algorithms on two medical imaging modalities. Such significant datasets were selected based on their application in diseases with high prevalence, morbidity and mortality. However, we acknowledge the limitation of not including many other forms of medical imaging. In future work, it is pertinent to explore the influence of different input data types, for example, chest X-ray, color fundus photography, computed tomography, and magnetic resonance imaging.

## 8. Related work

With a growing interest in have been many approaches proposed in the literature. This has been followed by several surveys and empirical papers that aim to provide an overview of the field and evaluate the performance of these methods. To enable a structured comparison between continual learning methods, Van de Ven and Tolias (6) described the three scenarios of learning. Parisi et al. (41) discussed continual learning from the perspective of biological lifelong learning such as structural plasticity, memory replay, curriculum and transfer learning etc. Delange et al. (42) offered a comprehensive experimental comparison of 11 different continual learning methods, however, they focused on easier task incremental setting and assume that the task identifier is known at the time of inference. In their interesting work Farquhar and Gal (43) examined standard evaluation practices and observed that based on the selection of experimental design, some continual learning approaches look better than they are. Qu et al. (44) grouped continual learning methods by their representative techniques, including regularization, knowledge distillation, memory, generative replay, parameter isolation etc. The study conducted by Hayes et al. (45) presents a thorough comparison between replay in the mammalian brain and replay in artificial neural networks and identified the gaps between replay in these two fields. Most of the empirical surveys (46–48) cover all three scenarios of continual learning and select only a handful of approaches suitable for each scenario for comparing performances. Hence, these works do not deep dive into a more focused use case of exemplar-free class-incremental setting which is particularly relevant to the medical domain. Furthermore, they do not compare these state-of-the-art algorithms on medical datasets. Research conducted by Derakhshani et al. (49) is closest to our work, where the authors have established a benchmark for classifying diseases using the MedMNIST dataset. However, they have considered a limited selection of five continual learning methods across all three scenarios of continual learning. As exemplar-free methods, they chose EWC (8), MAS (11), and LwF (19), while iCarL (50) and EEIL (51) were selected as exemplar-based methods. They reported iCarL achieving the highest performance on the PathMNIST dataset with an accuracy of 58.46%. In contrast, EFT (29), an expansion-based exemplar-free method, performed the best (66.82%) on the PathMNIST dataset based on the current study.

## 9. Conclusion

Three major continual learning methods namely regularization-based, expansion-based, and generative replay-based methods, and relevant algorithms were explained and summarized in this research. Furthermore, twelve state-of-the-art privacy-preserving continual learning algorithms were investigated for medical imaging classification using deep learning models. BIR algorithm achieved the best average accuracy on OCT for retinal disease classification among all continual learning algorithms, and EFT is the best-performing algorithm on PathMNIST for colorectal cancer histology classification. It was suggested both expansion-based and generative replay-based methods, specifically EFT and BIR, show the greatest potential in continual learning for medical applications. Given the frequent model updates and the need for the integration of new medical knowledge, continual learning has become an increasingly important topic in healthcare model deployment. Nevertheless, the trade-off between performance loss and patient privacy remains a crucial consideration. Continual learning offers a promising avenue for improving model performance while preserving patient privacy, and could potentially be the next paradigm for next-generation deep learning-based medical image classification.

## Data availability statement

Publicly available datasets were analyzed in this study. OCT data can be found at: https://data.mendeley.com/datasets/rscbjbr9sj/3, PathMNIST data can be found at: https://zenodo.org/record/6496656#.ZGMJ--xByys and CIFAR10 data can be found at: https://www.cs.toronto.edu/~kriz/cifar.html.

## Author contributions

TV and LJ: conceptualization. TV, LJ, JZ, JH, MT, and BC: methodology, implementation, and experiments. TV, LJ, JZ, JH, MT, and TT: draft preparation and visualization. TV, LJ, FG, and XX: review and editing. DT and YL: supervision. All authors have read and agreed to the published version of the manuscript.
