# Supplementary material for: Privacy-preserving continual learning methods for medical image classification: a comparative analysis
Source: Front Med (Lausanne). 2023 Aug 14;10:1227515. doi: 10.3389/fmed.2023.1227515 (PMC10461441; doi:10.3389/fmed.2023.1227515)
Supplement: Supplementary file 1 [file Data_Sheet_1.PDF]

Supplementary Material

1 COMPARED METHODS

In this section we provide a succinct overview of the compared methods, emphasizing their key features, and potential limitations.

1.1 Finetune

In conventional deep learning, weights between different convolution layers are finetuned for the best output performance. Hence, it suffers from catastrophic forgetting when weights are optimized for new tasks without considering previous tasks' performance. Finetune approach is regarded as the lower bound.

1.2 Joint

In joint method, the model is trained using the accumulated data of the current task as well as all previous tasks, therefore, it achieves the best overall performance. This approach serves as the upper bound.

1.3 Learning without forgetting (LwF)

LwF (Li and Hoiem, 2017) is one of the typical data-based regularization algorithms and uses knowledge distillation to retain the knowledge of preceding tasks. Before training on the new task, the network's outputs for the new task data are recorded and are later employed during training to transfer prior task knowledge. In particular, LwF optimizes two loss functions, knowledge distillation loss to maintain stability, and cross-entropy loss to maintain plasticity. A well-known limitation of LwF is its strong dependence on the similarity between new and old tasks, which may result in poor performance when the distributions of the new and old tasks are vastly different.

1.4 Learning without Memorizing (LwM)

LwM (Dhar et al., 2019) is another data-based regularization algorithm. It adds another loss named attention distillation loss along with knowledge distillation loss and cross-entropy loss. The key idea behind this approach is that the attention map used by the model trained on previous tasks should remain stable during training on a new task, with the expectation that the features that contribute to a certain class label decision should remain constant. The attention map is computed using Grad-CAM (Selvaraju et al., 2017) algorithm.

1.5 Elastic weight consolidation (EWC)

EWC (Kirkpatrick et al., 2017) is a weight-based regularization method which penalizes changes in important previous tasks' parameters. The method works by keeping track of the Fisher Information Matrix (FIM) for each task. The FIM is an approximation of the local curvature of the loss function with respect to the parameters of the model. It captures the importance of each parameter for a given task by measuring how much the loss function changes when the parameter is perturbed. A drawback of EWC is that the assumption that the FIM is diagonal may not hold true in real-world scenarios.

1.6 Synaptic intelligence (SI)

Similar to EWC, SI (Zenke et al., 2017a) is another weight-based regularization approach. It defines both weights and biases between CNN layers as synapses. The importance of each individual synapse

is calculated by its performance improvement in past tasks. For new tasks, this algorithm regularizes by penalizing significant changes to synapse based on its importance. As new classes are learnt continuously, the forgetting is minimized by finding alternative synapses similar to the old classes' output.

1.7 Memory Aware Synapses (MAS)

Similar to other weight-based regularization methods such as EWC and SI, MAS (Aljundi et al., 2018) accumulates the importance of each parameter of the network. However, this algorithm computes the parameter weights by measuring the sensitivity of the prediction function with respect to the parameter's change, instead of the sensitivity of the loss of certain task (Zenke et al., 2017b), so it is possible to use this method in an unsupervised or online manner.

1.8 MUlti-Classifier Memory Aware Synapses (MUC-MAS)

On top of MAS algorithm, Liu et al. (2020) developed a multi-classifier paradigm which integrates auxiliary classifiers to achieve more effective regularization results. The key idea of MUC-MAS is to train multiple auxiliary classifiers using out-of-distribution data which are irrelevant to the current task. These classifiers are able to formulate different decision boundaries for the same classification objective, thus helping to generate complementary information that reduces the forgetting of learned categories in a regularisation manner.

1.9 Gradient Projection Memory (GPM)

GPM method (Saha et al., 2021) restricts the direction of gradient descent steps such that there is minimum interference with past tasks, thereby retaining the knowledge gained from past tasks while learning new tasks. More specifically, after each task, the gradient sub-spaces that are most important for past tasks are determined and the bases are extracted using Singular Value Decomposition (SVD) and stored in GPM. When learning the next task, the gradient steps taken are orthogonal to the space spanned by the GPM to minimise forgetting on past tasks.

1.10 Orthogonal Weights Modification (OWM)

In OWM algorithm (Zeng et al., 2019), the gradients from new task training are projected to the direction that is orthogonal to the subspace spanned by samples from previous tasks. As the training goes task by task, a representative vector P of the past feature space is iteratively computed for each layer and the gradients from the new task are projected to the direction orthogonal to P.

1.11 Riemannian Walk (RWalk)

RWalk (Chaudhry et al., 2018) is inspired by two continual learning algorithms, EWC and SI. Similar to EWC regularization, it introduces Riemannian manifold distance as an approximation to model likelihood distribution. The SI is modified to calculate parameter importance based on output difference in Riemannian manifold distance. However, it has been demonstrated, and as our research indicates, that RWalk by itself is insufficient to prevent catastrophic forgetting in certain scenarios.

1.12 Efficient Feature Transformations (EFT)

EFT (Verma et al., 2021) is one of the expansion-based algorithms which is exemplar-free and predicts task identifiers. It partitions the model into global parameters and task-specific local parameters. During training a task, only the task-specific parameters are trained; previous local parameters and global parameters remain unchanged. Also, to make the training more efficient, it uses groupwise and depthwise convolutions,

producing new features for a specific task. At inference time, EFT predicts task identifiers by selecting the task-specific set of parameters that produces the maximum confidence prediction for the input. It also uses feature distance maximization regularization to increase task prediction ability. As the network for EFT is ”multi-headed”, it generally performs better than other exemplar-free continual learning approaches in the class-incremental learning scenario.

1.13 Generative Replay (GR)

In the GR (Shin et al., 2017) framework, the model retains previously acquired knowledge by the concurrent replay of generated pseudo-data. Inspired by mammalian memory consolidation by information replay in the brain, GR builds a generative model concurrently. The mammalian brain evolves to use self-digested thoughts for replay instead of storing raw sensory input information. Similarly, a generative adversarial network based generative model learns from old task input and selects the most similar data for replay. During training on new tasks, generated data are interleaved with new data to update the model. Similarly, the generator network is also updated using pseudo-data and new data. The basic version of the GR method has been found to perform poorly on complex datasets.

1.14 Brain Inspired Replay (BIR)

BIR (van de Ven et al., 2020) proposes a new, brain-inspired variant of replay in which internal or hidden representations (instead of pixel-level image) are replayed that are generated by the network’s own, context-modulated feedback connections. Instead of having a separate network for generating pseudo-data, the generator is integrated into the main model by adding backward connections. BIR employs a variational autoencoder (VAE) architecture as the generator component. Furthermore, to enable the model to generate specific classes, the standard normal prior is replaced by a Gaussian mixture with a separate mode for each class. As we see in our work, BIR improves the performance of GR considerably.

2 NETWORK ARCHITECTURE AND HYPERPARAMETERS

We utilized the Avalanche library, developed by Lomonaco et al. (2021), for the Finetune, Joint, LwF, EWC, SI, and RWalk approaches. Avalanche is a Pytorch-based, end-to-end continual learning library that includes benchmarks, algorithms, and evaluation metrics for continual learning. For LwM we used the implementation provided by Zhong (2021) and for the remaining methods, we employed the official implementation supplied by the authors. The official implementation assumes equal number of classes in each task, but we modified it to handle unbalanced classes in our experiment. We used ResNet50 (He et al., 2016) as our backbone convolutional neural networks (CNN) architecture. RTX3090 was used for training.

We conducted a thorough search for optimal hyperparameters to achieve the best results in class incremental learning scenario. For Finetune, we set the learning rate as 1e-4 and for the remaining algorithms it was set to 1e-3 unless it is mentioned otherwise. The number of training epochs varied depending on the algorithm used. For most methods, we trained each task for 8 epochs on the OCT dataset and 64 epochs on the PathMNIST and CIFAR10 datasets, unless otherwise specified. Following are the detailed hyperparameters settings for each algorithm.

- LwF: The temperature was set to 1 and the learning rate was set to 1e-4. The coefficient of distillation loss was set to different values for each dataset. For OCT and CIFAR10 it was set to 1000 and for PathMNIST it was set to 100.

- LwM: After exploring the range of 0.1-5 for the hyperparameter, the coefficient for the knowledge distillation loss was determined to be 2 and the coefficient for the attention distillation loss was set at 0.5.
- EWC: The value of the coefficient parameter that yielded the best results for the OCT and PathMNIST datasets was 1e5, while it was set to 1e3 for the CIFAR10 dataset.
- SI: Strength parameter c (coefficient of regularization loss) and damping parameter ξ (used while computing regularization loss) are the two parameters that need to be tuned. Different combinations of these parameters were tested for each dataset. For OCT, $c = 6500$ and $\xi =$ 1e-7; for PathMNIST, $c = 15000$ and $\xi =$ 1e-7; and for CIFAR, $c = 4000$ and $\xi =$ 1e-5 were used.
- MAS/MUC-MAS: We set coefficient of regularization loss to 1e-2 for both the methods.
- GPM: We tuned learning rate and number of epochs for each task. We observed that setting epochs to a higher values resulted into model forgetting about previous tasks, hence we used epochs between 5-20 for different datasets. The learning rates used varied between 1e-5-1e-3.
- OWM: For CIFAR10, the model was trained for 20 epochs with a learning rate of 2e-2 per task. However, adaptive learning rates and epochs were required for OCT and PathMNIST. OCT tasks were trained for 5 epochs with learning rates of 2e-4 and 2e-9. PathMNIST's first task had 5 epochs with a learning rate of 2e-2, the second had 50 epochs with 5e-1, and the final task had 500 epochs with 1e7.
- RWalk: The value of λ (coefficient for regularization loss) was set to 1e3 for all datasets. For the OCT and MedMNIST datasets, α (moving average factor for the Fisher matrix) was set to 0.1 and Δt (interval for updating parameter scores) was set to 10, while for the CIFAR10 dataset, α was set to 0.9 and Δt was set to 50.
- EFT: We used 1e-2 as learning rate and the coefficient of the regularization loss was set to 0.05. We trained the model for 128 epochs.
- GR/BIR: Higher number of epochs were required for generative based methods. We trained for 1000 epochs on OCT and CIFAR10 datasets and 5000 epochs on PathMNIST dataset. Learning rate for the OCT and CIFAR10 datasets was set to 1e-3, and for PathMNIST it was set to 1e-4.

3 EXPERIMENTS

Given the highly unbalanced nature of OCT data, characterized by varying numbers of training samples in each class, we conducted additional investigations. These investigations explored the performances of the selected algorithm under different class sequences, including scenarios where task 1 contained a large amount of data while task 3 had the least amount of data, and vice versa.

Table S1 provides descriptions of three different combinations of classes for each task, along with the percentage of training data indicated in brackets. In Sequence 1 (same as used in the main paper), the initial task has a large number of training data compared to later tasks. In Sequence 2, the training data starts with a small amount, but later tasks have a large amount of training data. In Sequence 3, the second task has the lowest training data among the three tasks.

Table S2 presents the overall accuracy and forgetting of Model 3 for the three sequences, while Table S3 displays the task-wise accuracies. In Figures 1a, 1b, and 1c we show how overall accuracy is affected after the model is trained on each task sequentially for the three sequences. Please note that the discrepancy in accuracy for the joint method is due to computing the average accuracy task-wise, not class-wise. LWM, SI, GPM, OWM and RWalk algorithms are found to be sensitive to the amount of training data

available for each task. Although their overall accuracies are comparable across the three sequences, their task-wise accuracy varies significantly. For instance, GPM manages to retain accuracy for the first task in sequence 1 but experiences complete forgetting in the other two sequences. Similarly, SI's accuracy for the last task improves when more training data is available. Likewise, RWalk's performance improves (sequence 2 and 3) when the amount of training data is not highly imbalanced across tasks. The remaining regularization-based methods and GR are largely unaffected by the change in class sequence, maintaining similar average accuracy and task-wise accuracy trends. While EFT offers nearly similar overall accuracy, its task-wise accuracy varies for the three sequences. Additionally, we observed an increase in forgetting when earlier tasks had a smaller amount of training data. The generative-based method BIR demonstrated robustness to the amount of training data available for each task. It maintained consistent performance across the sequences concerning overall accuracy, forgetting, and task-wise accuracy. In conclusion, BIR is better suited for scenarios with an imbalance in the amount of training data available for each task.

REFERENCES

Aljundi, R., Babiloni, F., Elhoseiny, M., Rohrbach, M., and Tuytelaars, T. (2018). Memory aware synapses: Learning what (not) to forget. In *Proceedings of the European Conference on Computer Vision (ECCV)*

Chaudhry, A., Dokania, P. K., Ajanthan, T., and Torr, P. H. (2018). Riemannian walk for incremental learning: Understanding forgetting and intransigence. In *Proceedings of the European Conference on Computer Vision (ECCV)*. 532–547

Dhar, P., Singh, R. V., Peng, K.-C., Wu, Z., and Chellappa, R. (2019). Learning without memorizing. In *Proceedings of the IEEE/CVF conference on computer vision and pattern recognition*. 5138–5146

He, K., Zhang, X., Ren, S., and Sun, J. (2016). Deep residual learning for image recognition. In *Proceedings of the IEEE conference on computer vision and pattern recognition*. 770–778

Kirkpatrick, J., Pascanu, R., Rabinowitz, N., Veness, J., Desjardins, G., Rusu, A. A., et al. (2017). Overcoming catastrophic forgetting in neural networks. *Proceedings of the national academy of sciences* 114, 3521–3526

Li, Z. and Hoiem, D. (2017). Learning without forgetting. *IEEE transactions on pattern analysis and machine intelligence* 40, 2935–2947

Liu, Y., Parisot, S., Slabaugh, G., Jia, X., Leonardis, A., and Tuytelaars, T. (2020). More classifiers, less forgetting: A generic multi-classifier paradigm for incremental learning. In *Computer Vision – ECCV 2020*, eds. A. Vedaldi, H. Bischof, T. Brox, and J.-M. Frahm (Cham: Springer International Publishing), 699–716

Lomonaco, V., Pellegrini, L., Cossu, A., Carta, A., Graffieti, G., Hayes, T. L., et al. (2021). Avalanche: an end-to-end library for continual learning. In *Proceedings of IEEE Conference on Computer Vision and Pattern Recognition*. 2nd Continual Learning in Computer Vision Workshop

Saha, G., Garg, I., and Roy, K. (2021). Gradient projection memory for continual learning. *arXiv preprint arXiv:2103.09762*

Selvaraju, R. R., Cogswell, M., Das, A., Vedantam, R., Parikh, D., and Batra, D. (2017). Grad-cam: Visual explanations from deep networks via gradient-based localization. In *Proceedings of the IEEE international conference on computer vision*. 618–626

Shin, H., Lee, J. K., Kim, J., and Kim, J. (2017). Continual learning with deep generative replay. *Advances in neural information processing systems* 30

van de Ven, G. M., Siegelmann, H. T., and Tolias, A. S. (2020). Brain-inspired replay for continual learning with artificial neural networks. *Nature communications* 11, 1–14

Verma, V. K., Liang, K. J., Mehta, N., Rai, P., and Carin, L. (2021). Efficient feature transformations for discriminative and generative continual learning. In *Proceedings of the IEEE/CVF Conference on Computer Vision and Pattern Recognition*. 13865–13875

Zeng, G., Chen, Y., Cui, B., and Yu, S. (2019). Continual learning of context-dependent processing in neural networks. *Nature Machine Intelligence* 1, 364–372

Zenke, F., Poole, B., and Ganguli, S. (2017a). Continual learning through synaptic intelligence. In *International Conference on Machine Learning* (PMLR), 3987–3995

Zenke, F., Poole, B., and Ganguli, S. (2017b). Continual learning through synaptic intelligence. In *Proceedings of the 34th International Conference on Machine Learning*, eds. D. Precup and Y. W. Teh (PMLR), vol. 70 of *Proceedings of Machine Learning Research*, 3987–3995

[Dataset] Zhong, C. (2021). Implementation of continual learning methods. `https://github.com/zhchuu/continual-learning-reproduce`

TABLES

Table S1. Different sequence of classes with corresponding training data distribution.

Sequence	Task 1	Task 2	Task 3
Sequence 1	Normal, CNV (81.56%)	DME (10.48%)	Drusen (7.96%)
Sequence 2	Drusen, DME (18.44%)	CNV (34.35%)	Normal (47.21%)
Sequence 3	Normal, Drusen (55.17%)	DME (10.48%)	CNV (34.35%)

Table S2. Average accuracy and forgetting of Model 3 for the three sequences.

Category	Method	Sequence 1		Sequence 2		Sequence 3	
		Accuracy	Forgetting	Accuracy	Forgetting	Accuracy	Forgetting
Baseline	Finetune	33.33	100	33.33	99.48	33.33	99.52
	Joint	90.76	-	95.2	-	95.07	-
Regularization	LwF	44.8	80.23	38.6	89.48	35.57	94.36
	LwM	41.75	41.58	40.38	24.99	34.98	67.74
	EWC	31.54	76.83	32.18	95.82	32.96	97.63
	SI	43.60	**21.27**	33.78	92.28	39.93	79.98
	MAS	31.73	83.69	34.02	76.29	30.31	81.03
	MUC-MAS	39.32	76.87	39.96	71.09	36.19	76.91
	GPM	41.16	26.41	33.33	95.76	33.33	75.47
	OWM	38.93	83.10	27.87	**20.1**	32.47	**19.44**
	RWalk	33.33	100	39.58	99.73	42.67	88.1
Expansion	EFT	43.20	38.13	41.27	62.23	43.26	74.18
Generative	GR	35.83	66.05	33.87	84.01	31.73	62.9
	BIR	**62.00**	51.31	**59.8**	55.6	**52.24**	61.17

Table S3. Task wise accuracy on Model 3 for the three sequences.

Category	Method	Sequence 1			Sequence 2			Sequence 3		
		Task 1	Task 2	Task 3	Task 1	Task 2	Task 3	Task 1	Task 2	Task 3
Baseline	Finetune	0.0	0.0	100	0.0	0.0	100	0.0	0.0	100
	Joint	99.47	97.47	75.33	86.4	99.6	99.6	86.4	99.2	99.6
Regulari-	LwF	0.53	39.33	94.53	0.0	19.3	96.5	0.0	9.23	97.47
-zation	LwM	63.13	0.93	61.20	58.87	14.93	47.33	9.6	0.0	95.33
	EWC	0.01	0.13	94.47	0.0	1.0	95.53	0.0	1.93	96.93
	SI	63.33	14.53	52.93	1.3	0.0	100	19.13	3.33	97.33
	MAS	0.13	10.37	84.68	6.07	15.14	80.86	4.41	12.82	73.71
	MUC-MAS	9.28	20.99	87.69	12.18	22.0	85.68	13.54	14.14	80.83
	GPM	90.66	1.73	31.07	0.0	0.0	100	0.0	0.0	100
	OWM	14.40	13.60	88.80	47.6	25.6	10.4	50.87	22.0	24.53
	RWalk	0.0	0.0	100	6.47	12.27	100	9.6	18.4	100
Expansion	EFT	61.47	38.40	29.73	16.73	47.33	54.0	15.77	34.8	79.2
Generative	GR	2.57	16.81	88.11	1.27	25.8	74.53	2.8	29.2	63.2
	BIR	10.53	78.40	97.07	6.67	73.33	99.4	6.33	52.8	97.6

FIGURE CAPTIONS

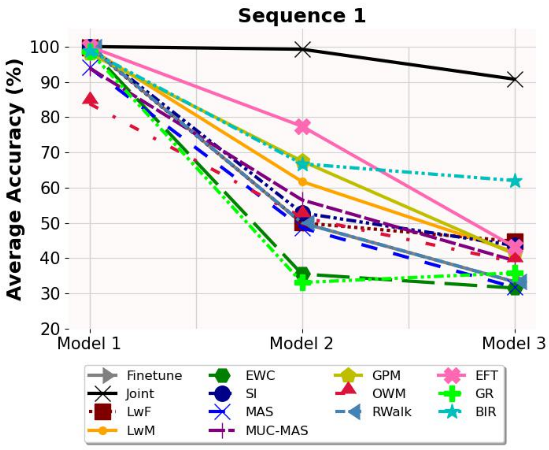

Figure 1a. Average accuracy for Sequence 1.

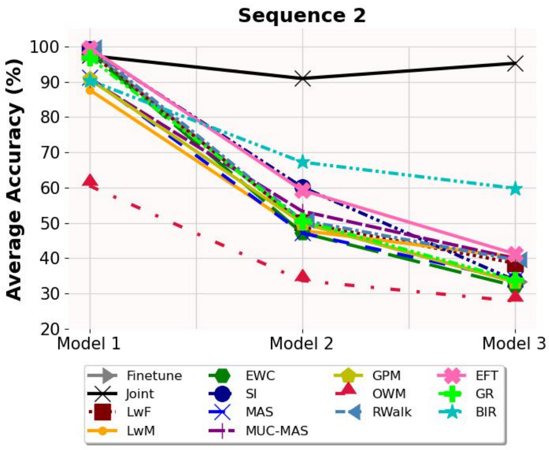

Figure 1b. Average accuracy for Sequence 2.

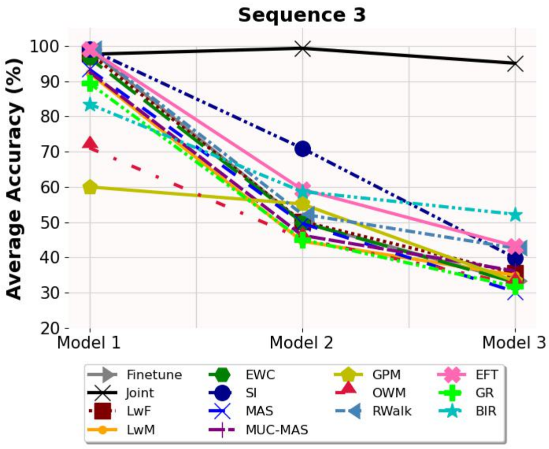

Figure 1c. Average accuracy for Sequence 3.

Figure 1. The average accuracy for the three sequences on the three models.
